# In Vitro Antimicrobial Activity of *Thymus vulgaris*, *Origanum vulgare*, *Satureja montana* and Their Mixture against Clinical Isolates Responsible for Canine Otitis Externa

**DOI:** 10.3390/vetsci10010030

**Published:** 2023-01-01

**Authors:** Valentina Virginia Ebani, Ylenia Pieracci, Giulia Cagnoli, Fabrizio Bertelloni, Chiara Munafò, Simona Nardoni, Luisa Pistelli, Francesca Mancianti

**Affiliations:** 1 Department of Veterinary Sciences, University of Pisa, 56124 Pisa, Italy; 2 Interdepartmental Research Center “Nutraceuticals and Food for Health” (NUTRAFOOD), University of Pisa, 56121 Pisa, Italy; 3 Department of Pharmacy, University of Pisa, 56121 Pisa, Italy

**Keywords:** essential oil, *Origanum vulgare*, *Satureja montana*, *Thymus vulgaris*, antibacterial activity, antifungal activity, canine otitis

## Abstract

**Simple Summary:**

Otitis externa of dogs is the inflammation of the external ear canal and may be acute or chronic, persistent or recurrent. Several bacterial species are involved in otitis externa and often *Malassezia* yeasts are simultaneously present. Otitis externa is often a severe problem in veterinary medicine because of the resistance of the involved pathogens to conventional drugs. Essential oils (EOs) could be promising products with which to treat these inflammations. EOs from *Origanum vulgare, Satureja montana,* and *Thymus vulgaris* seem to be active to the main bacterial species and *M. pachydermatis* cultured from the ears of dogs with otitis; moreover, a mixture of these three components seems to improve the antibacterial property.

**Abstract:**

Otitis externa is a frequent inflammation among dogs, mainly caused by bacteria and yeasts that are often resistant to conventional drugs. The aim of the present study was to evaluate the in vitro antibacterial and antifungal activities of commercial essential oils (EOs) from *Origanum vulgare, Satureja montana,* and *Thymus vulgaris,* as well as a mixture of these three components, against 47 clinical bacterial strains (*Staphylococcus* sp., *Streptococcus* sp., *Pseudomonas aeruginosa*, *Escherichia coli*, *Klebsiella pneumoniae*, *Serratia marcescens*) and 5 *Malassezia pachydermatis* strains, previously cultured from the ears of dogs affected by otitis externa. The tested Gram-positive bacteria were sensible to the analysed EOs with MICs ranging from 1.25% (*v*/*v*) to <0.0195% (*v*/*v*); Gram-negative isolates, mainly *P. aeruginosa,* were less sensitive with MICs from >10% (*v*/*v*) to 0.039% (*v*/*v*). *M. pachydermatis* isolates were sensitive to all EOs with MICs from 4.25% (*v*/*v*) to 2% (*v*/*v*). However, the mixture was active against all bacterial (except one *P. aeruginosa* strain) and fungal tested isolates. The three EOs and their mixture seem to be an interesting alternative for treating canine otitis externa when conventional antimicrobials are not active.

## 1. Introduction

Otitis externa of dogs is the inflammation of the external ear canal, including the ear pinna, and may be acute or chronic, persistent or recurrent. Changes occur in the canine external ear canal with otitis such as glandular hyperplasia, glandular dilation, epithelial hyperplasia, and hyperkeratosis, which cause increased cerumen production. This condition, characterized by higher local humidity and pH, predisposes the ear to secondary infection [1]. The bacteria most commonly isolated from ear canals of dogs affected by otitis are *Staphylococcus* spp., but other bacterial species, including *Streptococcus* spp., *Pseudomonas* spp., *Escherichia coli* and other Enterobacteriaceae may be involved, too [1,2,3]. *Malassezia* yeast is another frequent agent encountered in canine otitis externa. The frequency of infection as sole causative agent is reported between 8% and 26% [4,5]. Some dogs appear to develop an allergic response to *Malassezia* spp., leading to significant discomfort and pruritus [1] and, when not properly treated the affection can evolve in otitis media, as well [6]. Therefore, an effective therapy, together with the correction of predisposing factors and concurrent diseases, is mandatory to prevent relapses [7]. 

Topical antimicrobial therapy is preferred to systemic treatment in case of otitis externa, but frequently it is not successful because of the resistance of bacteria and yeast agents to conventional drugs. Therefore, essential oils (EOs) are welcome as alternative therapies and studies about their effectiveness against bacteria and yeasts responsible for otitis externa are necessary. 

EOs from oregano (*Origanum vulgare* L.), savory (*Satureja montana* L.) and thyme (*Thymus vulgaris* L.) showed in vitro antimicrobial properties when tested against bacterial and fungal species [8], but specific information about their effectiveness against clinical strains isolated from dogs affected by otitis are very scanty [3].

The present study focused on investigating the in vitro antibacterial and antifungal activities of three commercial EOs versus clinical strains previously isolated from the ears of dogs affected by otitis externa. In particular, EOs from *O. vulgare, S. montana,* and *T. vulgaris*, as well as a mixture of these three components, were tested against isolates of *Staphylococcus* sp., *Streptococcus* sp., *Pseudomonas aeruginosa*, *E. coli*, *Klebsiella pneumoniae*, *Serratia marcescens,* and *Malassezia pachydermatis*. 

## 2. Materials and Methods 

### 2.1. Essential Oils 

Three commercial EOs, obtained from oregano (*Origanum vulgare* L. subsp. *hirticum*), savory (*Satureja montana* L.), and thyme (*Thymus vulgaris* L.), were employed in the study. The EOs were obtained from FLORA^®^ (Pisa, Italy). A mixture was prepared with equal parts of the three oils. All EOs and the mixture were kept at 4 °C in dark glass vials; before being used in the analyses, they were microbiologically tested for quality control. 

### 2.2. Gas Chromatography—Mass Spectrometry Analysis 

The selected EOs were diluted to 5% in HPLC-*grade n*-hexane before the injection. The Gas Chromatography–Mass Spectrometry (GC-MS) analyses were carried out following the protocol previously reported by Pieracci et al. [9]. Agilent 7890B gas chromatograph (Agilent Technologies Inc., Santa Clara, CA, USA) equipped with an Agilent HP-5MS capillary column (30 m × 0.25 mm; coating thickness 0.25 μm) and an Agilent 5977B single quadrupole mass detector was used. The analytical conditions were the following: oven temperature increasing from 60 to 240 °C at 3 °C/min; injector and transfer line temperatures set at 220 °C and 240 °C, respectively; carrier gas helium flow set at 1 mL/min. The injection volume was 1 μL, with a split ratio of 1:25. The acquisition parameters were: full scan; scan range: 30–300 *m*/*z*; scan time: 1.0 s. The identification of the constituents was based on a comparison of the retention times with those of the authentic samples, comparing their linear retention indices relative to the series of n-hydrocarbons. Computer matching was also used against commercial (NIST 14 and ADAMS 2007) and laboratory-developed mass spectra libraries built up from pure substances and components of commercial essential oils of known composition and MS literature data [10,11,12,13,14,15].

### 2.3. Antibacterial Activity 

#### 2.3.1. Bacterial Strains 

Forty-seven bacterial strains, 23 Gram positive (22 strains of *Staphylococcus* spp., 1 of *Streptococcus constellatus*) and 24 Gram negative (16 strains of *Pseudomonas aeruginosa*, 6 of *Escherichia coli*, 1 of *Serratia marcescens*, 1 of *Klebsiella pneumoniae*) were tested with the three EOs and the mixture. 

All strains were previously cultured from ears of dogs affected by otitis externa. After isolation, the strains were typed using API Staph, API Strep, API 20E, and API 20NE systems (BioMerieux, Milan, Italy) in relation to microbiological and Gram staining characters. To discriminate between *S. aureus* and *S. pseudointermedius*, a multiplex-PCR assay described by Sasaki et al. [16] was employed.

The strains were stored at −80 °C in glycerol broth. Before being employed in the antibacterial activity analyses, each isolate was cultured in brain hearth infusion broth (BHIB, Oxoid Ltd., Basingstoke, Hampshire, UK) at 37 °C for 24 h. Cultures of 1–2 × 10^7^ CFU/mL, corresponding to 0.5 McFarland standard, were used in the tests.

#### 2.3.2. Antimicrobial Sensitivity Test 

The disc diffusion method (EUCAST, The European Committee on Antimicrobial Susceptibility Testing, disk diffusion method for anti-microbial susceptibility testing version 6.0) was used to verify the resistance to the following antimicrobials (Oxoid): penicillins (amoxycillin and clavulanic acid, AMC, 20–10 µg; ampicillin, AMP, 10 µg), fluoroquinolones (enrofloxacin, ENR, 5 µg), aminoglycosides (amikacin, AK, 30 µg; gentamicin, CN, 10 µg; tobramycin, TOB, 10 µg), lincosamides (clindamycin, DA, 2 µg), ansamycins (rifampicin, RD, 30 µg), folate pathway antagonist (trimethoprim–sulfamethoxazole, SXT, 19:1, 25 µg), and tetracyclines (tetracycline, TE, 30 µg; doxycycline, DO, 30 µg). The antimicrobials were selected on the basis of the antibiotic panel most frequently used in dogs by veterinarians. The test was carried out on Mueller–Hinton agar plates (Oxoid) incubated at 35 °C for 16–20 h; the results were interpreted on the basis of the breakpoints reported by EUCAST or CLSI (The Clinical and Laboratory Standards Institute) [17,18]. 

#### 2.3.3. EOs and Mixture Minimum Inhibitory Concentration 

In order to verify the sensitivity of the bacterial strains to the three EOs and their mixture, each bacterial isolate was tested to determine the minimum inhibitory concentration (MIC) through the broth microdilution method, according to the guidelines reported by CLSI [19] and the protocol reported by Ebani et al. [20]. The MIC value was the lowest concentration, expressed in percentage (10%, 5%, 2.5%, 1.25%, 0.625%, 0.312%, 0.156%, 0.078%, 0.039%, 0.0195% (*v*/*v*)), of each EO and mixture at which bacteria show no visible growth. The test was executed simultaneously to control bacterial growth (tested strain and media) and sterility (tested EO and media). All tests were executed in triplicate. 

### 2.4. Antifungal Activity 

Five *M. pachydermatis* clinical strains were analyzed to verify their in vitro antifungal sensitivity through microdilution method. The test was assessed using liquid m-Dixon medium for preparing yeast suspensions, as previously described [21]. The isolates were cultured from dogs affected by external otitis onto Mycobiotic agar (Thermo Fisher Scientific, Rodano, Italy). The fungal isolates were tested against ketoconazole by *E*-test (AB Biodisk, Solna, Sweden) to verify their susceptibility to this antimycotic drug, frequently used to treat *Malassezia* infections [22]. The fungal strains were analyzed in triplicate versus EOs and mixture diluted into the medium at concentrations of 10%, 5%, 4.5%, 4%, 3.75%, 3,5%, 3, 25%, 3%, 2.75; 2.5%, 2.25%, 2%, 1.5%, and 1%. MIC was determined as the lowest EO/mixture concentration where no fungal growth was observed.

## 3. Results

### 3.1. Gas Chromatography—Mass Spectrometry Analysis

The complete compositions of the analyzed commercial EOs and their mixture are reported in Table 1. Overall, 43 compounds were identified, covering 99.1–100.0% of the whole compositions. 

The mixture was characterized by oxygenated monoterpenes as the main chemical class of compounds, accounting for 75.0%, mainly represented by carvacrol (50.8%) and thymol (17.1%). Carvacrol was found in very considerable percentages in the EOs of *O. vulgare* and *S. montanta*, where it constituted 78.0 and 60.0% of the whole compositions, respectively. The EO of *T. vulgaris*, instead, showed a much lower content of this component (5.5%), but a higher amount of thymol (43.6%), responsible for the great content of this molecule in the mixture.

Monoterpene hydrocarbons were the second most represented class in the mixture sample, reaching 14.0%. *p*-Cymene, with 9.2%, was the most abundant compound belonging to this class, and probably derived from the EO of *T. vulgaris*, in which it covered 18.7% of the composition. However, it was found in not negligible percentages also in *O. vulgare* (3.3%) and *S. montana* (5.7%) EOs.

Finally, sesquiterpenes in either their hydrocarbon or oxygenated forms were also detected in the mixture, as well as in the individual EOs. β-Caryophyllene and its oxide were the most representative molecules belonging to these classes, and their content in the mixture and in the singular EOs was very similar.

### 3.2. Antibacterial Activity

#### 3.2.1. Antimicrobial Sensitivity Tests

The 22 analyzed staphylococci were resistant from 0 to 10 out of the 11 tested antimicrobials and showed 20 different resistance profiles (Table S1). The most effective antimicrobials were amoxicillin/clavulanic acid (86.36% susceptible isolates), trimethoprim/sulfamethoxazole (77.27% susceptible isolates), and amikacin (72.73% susceptible isolates). Most of the tested staphylococci were resistant to ampicillin (81.81%) and tobramycin (68.18%). *Streptococcus constellatus* was resistant only to tetracycline.

As regards bacteria of Enterobacteriaceae family, seven different resistance profiles were determined (Table S2). Analyzed bacterial strains were resistant from three to ten of the tested antimicrobials. The most effective antimicrobials were aminoglycosides and enrofloxacin, whereas 50.00% of tested isolates were resistant to doxycycline, tetracycline, and trimethoprim/sulfamethoxazole. More than 50% of tested *E. coli* were resistant to ampicillin. All Enterobacteriaceae were intrinsically resistant to clindamycin and rifampicin, *S. marcescens* is intrinsically resistant to ampicillin and amoxicillin/clavulanic acid, and *K. pneumoniae* is intrinsically resistant to ampicillin; obtained data are in line with these statements.

*Pseudomonas aeruginosa* isolates showed 11 different antimicrobial resistance profiles and resulted in resistance from 5 to 11 of the tested molecules (Table S3). *Pseudomonas aeruginosa* bacteria are intrinsically resistant to ampicillin, amoxicillin/clavulanic acid, tetracycline, trimethoprim/sulfamethoxazole, clindamycin, and rifampicin. This is confirmed by our data. Considering the remaining antimicrobials tested, tobramycin was the most effective (75.00% of susceptible isolates), whereas high resistance was detected for enrofloxacin (68.75%).

#### 3.2.2. EOs and Mixture Minimum Inhibitory Concentration

MIC values showed antibacterial properties of the selected EOs and their mixture against almost all tested isolates. The growth of Gram-positive strains was inhibited by all EOs and the mixture. In detail, MIC obtained with *O. vulgare* EO ranged from <0.0195% to 0.156%, with *S. montana* from 0.078% to 0.312%, with *T. vulgaris* from <0.0195% to 0.625%. The mixture gave the lowest MIC values: 12/23 tested strains had MIC <0.0195%, 8/23 strains had MIC 0.039%, 3/23 had MIC 0.078% (Table 2).

Gram-negative isolates were less sensitive to the employed EOs, when compared to the tested Gram-positive strains. In fact, one *P. aeruginosa* strain was resistant to *O. vulgare*, three to *S. montana*, and ten to *T. vulgaris*, as well as one *E. coli* strain to *T. vulgaris* and one *P. aeruginosa* to the mixture. Detected MIC values ranged from 0.039% to 0.625% with *O. vulgare*, from 0.039% to 2.5% with *S. montana*, from 0.156% to 5% with *T. vulgaris*. The mixture was more active with MICs < 0.0195% (3/6 *E. coli* isolates, 1/1 *K. pneumoniae* isolate), 0.039% (2/6 *E. coli*, 1/16 *P. aeruginosa*, 1/1 *S. marcescens* strains), 0.078% (1/6 *E. coli*, 1/16 *P. aeruginosa* strains), 0.156% (4/16 *P. aeruginosa* strains), 0.312% (3/16 *P. aeruginosa* strains), 0.625% (2/16 *P. aeruginosa* strains), 1.25% (2/16 *P. aeruginosa* strains), 5% (2/16 *P. aeruginosa* strains); only one *P. aeruginosa* isolate was resistant (Table 3).

Considering the bacterial species, one strain of *E. coli* (856A1) was resistant to *T. vulgaris,* and it was inhibited by *O. vulgaris* with 1.25% MIC value, but lowest MICs (0.039%) were obtained with *S. montana* and the mixture. The remaining five *E. coli* isolates were sensible to the three EOs, even though they showed the lowest MICs when assayed with the mixture. Both *K. pneumoniae* and *S. marcescens* were sensible to the three EOS and lower MICs were observed with the mixture (<0.0195% and 0.039%, respectively).

Different results were obtained when testing *P. aeruginosa* isolates in relation to EOs and bacterial strain. One isolate was resistant to *O. vulgare*, three to *S. montana* and ten to *T. vulgaris*. Except for isolate 535A, all strains were inhibited by the mixture.

### 3.3. Antifungal Activity

All isolates were sensible to ketoconazole. *Satureja montana* EO appeared as the more active compound, with MIC values of 2% in four out of five fungal isolates, followed by the other EOs with similar results. Interestingly, MICs of the mixture appeared slightly higher than those showed by *S. montana,* suggesting a possible antagonistic effect of the three oils, although the mixture was more effective with respect to *O. vulgare* and *T. vulgaris* alone (Table 4). 

## 4. Discussion

The findings of the present study showed antimicrobial properties of commercial EOs from *O. vulgare, S. montana,* and *T. vulgaris* against Gram-positive and Gram-negative bacteria, and *M. pachydermatis*, even though differences were observed in relation to the bacterial or fungal isolate and the tested EO.

Members of *Staphylococcus* genus are Gram-positive bacteria acting as mammalian commensals that can colonize mucosal membrane, nares, skin, and ears. Different staphylococcal species are frequently isolated from the ears of dogs affected by otitis. This is difficult to resolve because of resistance of these bacteria to many antibiotics. Resistance to methicillin and other antimicrobials are a spread threat with severe implications on animal and human health [23]. The tested staphylococcal isolates were resistant to several antibiotics and most of them were resistant to ampicillin (81.81%) and tobramycin (68.18%) in accordance with other surveys [23,24,25,26,27,28].

Oregano, savory, and thyme EOs were active against the tested *Staphylococcus* isolates in agreement with data reported by other investigations [3,29,30,31,32,33]. Low MIC values were found when testing the isolates against the three EOs; moreover, the sensitivity of *Staphylococcus* spp. strains increased (<0.0195% *v*/*v* in 11/22 isolates) when they were assayed with the mixture.

One strain of *S. constellatus* has been evaluated in this survey. Unfortunately, only one streptococcal isolate was available, therefore it is difficult to verify the real sensitivity of streptococci against the EOs. However, the tested strain was resistant to tetracycline, but wassensitive to oregano, thyme, and savory EOs and their mixture (MIC < 0.0195%). Data about the activity of EOs against this bacterial species are not present in the literature, but in previous studies *Origanum compactum*, *T. vulgaris*, *S. montana*, as well as *Cinnamomum verum* and *Cymbopogon citratus*, were active against a human strain of *Streptococcus pyogens* [34].

*Pseudomonas aeruginosa* is a Gram-negative opportunistic pathogen able to infect several tissues, including skin and the external ear of pet animals. It is frequently encountered in human hospital-acquired infections, and it has often been found in wastewaters [35]. The natural resistance of this bacterium and the large circulation of multidrug-resistant strains result in the failure of antibiotic therapies in human and veterinary medicine. The 16 *P. aeruginosa* isolates tested in this survey showed a high level of antibiotic-resistance; these results highlighted that *P. aeruginosa* strains often represent a serious issue for the health status of dogs for which it is difficult to find an appropriate therapy.

Previous studies investigated different EOs to detect natural antimicrobials with activity against *P. aeruginosa* antibiotic-resistant strains [35,36,37]. Recently, Van et al. [35] observed antibacterial activity of the thyme EO against *P. aeruginosa* multidrug-resistant isolates, cultured from human clinical samples and wastewaters. Similar observations have been reported in other investigations. It was observed that red thyme EO was more active against biofilm cells than their planktonic counterparts of both *P. aeruginosa* and *Pseudomonas putida* [38]. Moreover, Pandur et al. [39] found a good antioxidant activity of *T. vulgaris* EOs obtained at different phonologic phases of the plant. In particular, they observed that these EOs and thymol increased catalase and superoxide dismutase activity as well as the antioxidant capacity of the THP-1 macrophages.

*Escherichia coli* is a Gram-negative bacterium of Enterobacteriaceae family acting as commensal or pathogen in humans and animals. In dogs, it can cause not only intestinal infections, but also infections of genito-urinary tract, skin and external ear canal. *Escherichia coli* is intrinsically susceptible to the most frequently employed antimicrobials; however, this bacterial species is able to acquire resistance genes, mainly through horizontal gene transfer [40]. The isolates tested in this survey were resistant to two or more antibiotics, confirming that this species is often involved in the antibiotic-resistance issue.

Our results showed that the three selected EOs were active against the tested *E. coli* strains, except for the strain 856A1 that was not inhibited by *T. vulgaris*. The best results were obtained when testing *E. coli* isolates with the mixture; in fact, MIC values were generally lower than those obtained with the singular EOs. To the best of our knowledge, data regarding the activity of EOs against *E. coli* strains responsible for animal otitis externa are not available in the literature, thus our results are not easily compared to other studies. 

Previous papers evidenced the in vitro activity of some EOs against *E. coli* isolated from other sources. *Cinnamomum zeylanicum*, *O. vulgare*, *T. vulgaris*, and *Syzygium aromaticum* EOs showed antimicrobial effects when tested against an enteroinvasive *E. coli* strain [41]. Good activity of *O. vulgare* and *T. vulgaris* was also detected against multi-drug resistant *E. coli* strains isolated from canine urinary tract infections [42].

*Klebsiella pneumoniae* and *S. marcescens* are two species belonging to Enterobacteriaceae family, less frequently involved in otitis of animals. The two isolates tested in this study were resistant to different antimicrobials, showing that these bacterial species, even though not frequently involved in canine otitis cases, may be a serious threat for the choice of an effective antibiotic. Few studies about the activity of EOs against these bacteria have been performed [43,44,45,46], and no data about their effectiveness against strains isolated from cases of otitis are available. As a bacteria belonging to the same family of *E. coli*, and thus having a very similar bacterial cell wall, it is supposable that the same EOs that were active versus *E. coli* may inhibit *K. pneumoniae* and *S. marcescens*, as suggested by our results, which showed the sensitivity of the tested strains versus the three EOs and their mixture.

Our results highlighted sensitivity differences to EOs between Gram-positive and Gram-negative bacteria due to their different cell wall structure. Hydrophobic molecules can get into Gram-positive bacteria and act on the cell wall and cytoplasm, thanks to the cell wall structure [47,48]. Antimicrobial EOs are able to damage the cell wall and cytoplasmic membrane of bacteria, with consequent cell lysis and leakage of intracellular compounds [29]. However, EOs employed in this survey had good activity against all tested isolates and their antimicrobial properties were enhanced when they were used in the mixture.

MIC values of the selected EOs against *M. pachydermatis* appeared in agreement with the results of studies previously published [33,49]; however, the three EOs in combination did not yield any synergistic effect, suggesting the best efficacy of *S. montana* alone. 

The EO of *S. montana* were characterized by carvacrol and thymol as major detected compounds. The results partially agreed with different literature studies, all reporting a strong prevalence of the former compound, but also very noticeable amounts of *p*-cymene and a lower content of the oxygenated monoterpene thymol. This molecule, instead, was the most important one found in the analyzed *T. vulgaris* EO, whose chemical composition was partially in agreement with the findings observed by Najar et al. [50] for the “Thymol chemotype”, in which thymol was found as the most important compound, followed by *p*-cymene. However, the content of γ-terpinene, which in our work was totally absent, in the cited work reached good amounts, almost accounting for 5%. These findings were in agreement with our previous study [42], also reporting the chemical composition of the EO of *O. vulgare.* The cited work evidenced a prevalence of carvacrol and *p*-cymene, consistent with our results, but it did not report the presence of thymol, found, instead, in our sample.

## 5. Conclusions

EOs from *O. vulgare, S. montana* and *T. vulgaris* were generally active against the main bacterial species involved in otitis externa of dogs. Their in vitro antimicrobial properties were enhanced when they were combined in a mixture, inhibiting the bacterial growth at very low MIC values. Conversely, *M. pachydermatis* was more sensitive to *S. montana* EO alone, suggesting an antagonistic effect of the three EOs within the mixture.

Considering that otitis externa is a recurrent or persistent problem in many dogs, due to antimicrobial-resistant bacteria involved in the infections, and thus to the difficulty of finding an appropriate therapy, EOs of *O. vulgare, S. montana* and *T. vulgaris* could represent useful alternative remedies. Moreover, the use of a mixture containing the three oils in equal parts may inhibit the bacterial growth using very low EOs concentration. 

Previous surveys investigated the antimicrobial properties of oregano, thyme, and savory, but most of them were carried out on reference strains or few clinical isolates; furthermore, data about the sensitivity of bacterial or fungal strains of canine origin to these natural products are almost absent in the scientific literature. Our study gives data about the in vitro activity of oregano, thyme, and savory EOs and their mixture after testing several clinical isolates. The promising results, mainly those regarding the enhanced antibacterial effectiveness of the mixture, suggest the need to perform in vivo studies.

## Figures and Tables

**Table 1:** Relative percentage of the main constituents of the tested essential oils.

Compounds	l.r.i. ^1^	Class	Mixture	*Origanum vulgare*	*Satureja montana*	*Thymus vulgaris*
			Relative Abundance ± SD (%)			
α-pinene	933	mh	0.4 ± 0.03	0.2 ± 0.01	0.2 ± 0.00	0.6 ± 0.02
camphene	948	mh	0.3 ± 0.02	- ^2^	-	0.7 ± 0.01
1-octen-3-ol	977	nt	-	0.2 ± 0.00	0.3 ± 0.01	-
β-pinene	977	mh	0.3 ± 0.02	-	-	0.5 ± 0.01
myrcene	991	mh	0.3 ± 0.02	0.5 ± 0.01	0.2 ± 0.00	-
δ-3-carene	1011	mh	-	-	-	0.1 ± 0.00
α-terpinene	1017	mh	0.3 ± 0.01	0.3 ± 0.00	0.2 ± 0.01	0.1 ± 0.01
o-cymene	1022	mh	-	-	-	0.1 ± 0.01
p-cymene	1024	mh	9.2 ± 0.16	3.3 ± 0.11	5.7 ± 0.08	18.7 ± 2.11
limonene	1029	mh	1.1 ± 0.01	-	0.1 ± 0.01	3.1 ± 0.26
1,8-cineole	1031	om	1.4 ± 0.01	-	-	5.1 ± 0.09
γ-terpinene	1058	mh	1.7 ± 0.03	1.8 ± 0.03	2.5 ± 0.03	-
cis-sabinene hydrate	1066	om	0.1 ± 0.01	0.2 ± 0.02	0.1 ± 0.01	-
terpinolene	1089	mh	0.2 ± 0.00	-	-	0.4 ± 0.01
trans-sabinene hydrate	1098	om	-	0.1 ± 0.01	-	-
linalool	1101	om	0.7 ± 0.02	-	1.0 ± 0.04	1.2 ± 0.15
β-terpineol	1144	om	-	-	-	0.7 ± 0.08
isoborneol	1156	om	-	-	-	0.3 ± 0.03
borneol	1165	om	1.0 ± 0.04	0.2 ± 0.01	1.3 ± 0.04	1.9 ± 0.16
4-terpineol	1177	om	0.4 ± 0.02	0.5 ± 0.01	0.7 ± 0.02	-
p-cymen-8-ol	1185	om	0.2 ± 0.02	-	-	0.5 ± 0.06
α-terpineol	1191	om	1.0 ± 0.03	-	0.4 ± 0.01	3.6 ± 0.25
γ-terpineol	1197	om	0.3 ± 0.01	-	-	0.9 ± 0.10
nerol	1228	om	0.1 ± 0.02	-	-	0.5 ± 0.11
methyl carvacrol	1247	om	1.2 ± 0.03	-	3.1 ± 0.10	-
geraniol	1254	om	0.2 ± 0.01	-	-	0.7 ± 0.16
bornyl acetate	1286	om			0.1 ± 0.00	-
thymol	1292	om	17.1 ± 0.03	6.9 ± 0.03	9.5 ± 0.15	43.6 ± 0.07
carvacrol	1302	om	50.8 ± 1.26	78.0 ± 0.23	60.0 ± 0.5	5.5 ± 0.35
α-terpinyl acetate	1350	om	0.5 ± 0.05	-	-	2.0 ± 0.10
thymol acetate	1355	om	-	-	0.1 ± 0.01	-
carvacrol acetate	1376	om	-	-	0.2 ± 0.01	-
longifolene	1404	sh	-	-	-	0.2 ± 0.00
β-caryophyllene	1419	sh	4.1 ± 0.22	3.2 ± 0.21	4.3 ± 0.21	3.2 ± 0.02
α-humulene	1453	sh	0.3 ± 0.03	0.2 ± 0.01	0.1 ± 0.00	0.3 ± 0.02
viridiflorene	1495	sh	-	-	0.1 ± 0.01	-
β-bisabolene	1509	sh	0.9 ± 0.11	0.8 ± 0.03	1.4 ± 0.06	-
trans-γ-cadinene	1513	sh	-	-	0.1 ± 0.00	-
δ-cadinene	1524	sh	0.1 ± 0.02	-	0.3 ± 0.01	-
spathulenol	1577	os	-	-	0.2 ± 0.01	-
caryophyllene oxide	1582	os	5.2 ± 0.75	3.2 ± 0.10	7.1 ± 0.06	4.4 ± 0.75
humulene oxide II	1608	os	0.2 ± 0.04	0.1 ± 0.00	0.2 ± 0.01	0.2 ± 0.05
14-hydroxy-9-epi-(E)-caryophyllene	1670	os	0.2 ± 0.05	-	0.5 ± 0.03	
**Chemical classes**			**Mixture**	***Origanum vulgare***	***Satureja montana***	***Thymus vulgaris***
Monoterpene hydrocarbons (mh)			14.0 ± 0.08	6.4 ± 0.17	8.9 ± 0.13	24.3 ± 2.37
Oxygenated monoterpenes (om)			75.0 ± 1.11	85.9 ± 0.28	76.5 ± 0.21	66.5 ± 1.53
Sesquiterpene hydrocarbons (sh)			5.4 ± 0.37	4.2 ± 0.24	6.3 ± 0.29	3.7 ± 0.01
Oxygenated sesquiterpenes (os)			5.6 ± 0.83	3.3 ± 0.10	8.0 ± 0.03	4.6 ± 0.79
Other non-terpene derivatives (nt)			-	0.2 ± 0.00	0.3 ± 0.01	-
Total identified (%)			100.0 ± 0.01	100.0 ± 0.02	100.0 ± 0.01	99.1 ± 0.05

Legend. ^1^ Linear retention index on a HP 5-MS capillary column; ^2^ Not detected; mh: monoterpene hydrocarbons; om: oxygenated monoterpenes; sh: sesquiterpene hydrocarbons; os: oxygenated sesquiterpenes; nt: non-terpenes; pp: phenylpropanoids; od: oxygenated diterpenes.

**Table 2:** MIC values of the tested EOs and mixture expressed in percentage (*v*/*v*) against the selected Gram-positive isolates.

Identification Number of the Isolate	Bacterial Species	*Origanum vulgare*	*Satureja montana*	*Thymus vulgaris*	Mixture
T10	*Staphylococcus aureus*	0.156	0.156	0.312	0.039
T20	*Staphylococcus aureus*	0.078	0.156	0.156	<0.0195
T33 G	*Staphylococcus aureus*	0.078	0.156	0.312	<0.0195
T39	*Staphylococcus aureus*	0.078	0.039	0.312	<0.0195
T42	*Staphylococcus aureus*	0.039	0.039	0.078	<0.0195
248	*Staphylococcus aureus*	0.078	0.156	0.625	0.039
501	*Staphylococcus aureus*	0.078	0.156	0.312	0.078
387	*Staphylococcus aureus*	0.078	0.078	0.312	<0.0195
T15	*Staphylococcus auricularis*	0.078	0.156	0.312	0.039
T32	*Staphylococcus capitis*	0.156	0.156	1.25	<0.0195
530 A	*Staphylococcus capitis*	0.078	0.156	0.312	0.078
T11	*Staphylococcus chromogenes*	0.156	0.156	0.312	0.039
T3	*Staphylococcus epidermidis*	0.156	0.156	0.312	0.039
T22	*Staphylococcus epidermidis*	0.156	0.156	0.625	<0.0195
T26	*Staphylococcus epidermidis*	<0.0195	0.078	0.156	<0.0195
T28	*Staphylococcus hominis*	0.156	0.312	0.625	0.039
T53	*Staphylococcus pseudointermedius*	0.078	0.156	0.156	<0.0195
T33 P	*Staphylococcus lugdunensis*	0.312	0.156	0.625	0.039
T27	*Staphylococcus simulans*	<0.0195	< 0.0195	<0.0195	<0.0195
T31 G	*Staphylococcus simulans*	0.156	0.156	0.312	<0.0195
208 A	*Staphylococcus xylosus*	0.156	0.156	0.625	0.039
234 2A	*Staphylococcus xylosus*	0.078	0.156	0.625	0.078
T54	*Streptococcus constellatus*	0.039	0.078	0.078	<0.0195

**Table 3:** MIC values of the tested EOs and mixture expressed in percentage (*v*/*v*) against the selected Gram-negative isolates.

Identification Number of the Isolate	Bacterial Species	*Origanum vulgare*	*Satureja montana*	*Thymus vulgaris*	Mixture
33 B	*Escherichia coli*	0.078	0.156	0.625	0.039
198	*Escherichia coli*	0.078	0.156	0.625	<0.0195
502 B	*Escherichia coli*	0.156	0.078	0.625	0.078
856 A1	*Escherichia coli*	1.25	0.039	>10	0.039
856 B1	*Escherichia coli*	0.156	0.156	0.312	<0.0195
858 A	*Escherichia coli*	0.156	0.156	0.156	<0.0195
220 B	*Klebsiella pneumoniae*	0.156	0.156	1.25	<0.0195
178	*Pseudomonas aeruginosa*	0.156	>10	>10	0.156
348 B	*Pseudomonas aeruginosa*	0.625	>10	>10	0.312
389	*Pseudomonas aeruginosa*	1.25	>10	>10	0.312
417	*Pseudomonas aeruginosa*	0.625	2.5	5	0.625
465	*Pseudomonas aeruginosa*	0.312	0.312	>10	0.156
502 A	*Pseudomonas aeruginosa*	1.25	0.312	>10	0.156
535 A	*Pseudomonas aeruginosa*	0.312	2.5	>10	>10
768	*Pseudomonas aeruginosa*	0.312	1.25	1.25	0.078
822 A1	*Pseudomonas aeruginosa*	>10	2.5	>10	0.156
856 A2	*Pseudomonas aeruginosa*	0.625	1.25	2.5	0.312
856 B2	*Pseudomonas aeruginosa*	0.625	2.5	5	1.25
858 B	*Pseudomonas aeruginosa*	0.625	1.25	>10	1.25
875 B1	*Pseudomonas aeruginosa*	0.625	0.156	>10	0.625
876 A	*Pseudomonas aeruginosa*	2.5	0.625	>10	5
876 B2	*Pseudomonas aeruginosa*	0.312	0.156	0.312	0.039
1034	*Pseudomonas aeruginosa*	0.039	1.25	0.156	5
100	*Serratia marcescens*	0.156	0.156	0.625	0.039

**Table 4:** MIC values of the tested EOs and mixture expressed in percentage (*v*/*v*) against the selected Malassezia pachydermatis isolates.

Fungal Isolates	*Origanum* *vulgare*	*Satureja* *montana*	*Thymus* *vulgaris*	Mixture	Ketoconazole (μg)
*Malassezia pachydermatis* 1	4	2	4	3	0.02
*Malassezia pachydermatis* 2	3.75	2	4	3	0.04
*Malassezia pachydermatis* 3	4	2.25	4.25	3.5	0.02
*Malassezia pachydermatis* 4	4	2	4	3.25	0.02
*Malassezia pachydermatis* 5	4	2	4	3	0.02

## Data Availability

Data are reported in the manuscript.

## Supplementary Materials

The following supporting information can be downloaded at: https://www.mdpi.com/article/10.3390/vetsci10010030/s1, Table S1: Antimicrobial resistance profiles for each examined Gram-positive isolate; Table S2: Antimicrobial resistance profiles for each examined Enterobacteriaceae isolate. Table S3: Antimicrobial resistance profiles for each examined *Pseudomonas aeruginosa* isolate.
