# Supplementary material for: In Vitro Antimicrobial Activity of Thymus vulgaris, Origanum vulgare, Satureja montana and Their Mixture against Clinical Isolates Responsible for Canine Otitis Externa
Source: Vet Sci. 2023 Jan 1;10(1):30. doi: 10.3390/vetsci10010030 (PMC9864906; doi:10.3390/vetsci10010030)
Supplement: Supplementary file 1 [file vetsci-10-00030-s001.zip › vetsci-2082146-supplementary.pdf]

SUPPEMENTARY MATERIALS

Table S1. Antimicrobial resistance profiles for each examined Gram-positive isolate.

Identification number of the isolate	Bacterial species	Antimicrobial resistance profile
T10	*Staphylococcus aureus*	AMP
T20	*Staphylococcus aureus*	AMP, DA, DO,TE, TOB,SXT,ENR
T33 G	*Staphylococcus aureus*	AMP, DA, TE, TOB, AK, ENR
T39	*Staphylococcus aureus*	AMP, DA, DO, TOB, AK, RD
T42	*Staphylococcus aureus*	AMP, TOB, RD
248	*Staphylococcus aureus*	DO, TE, TOB, CN, SXT, ENR
501	*Staphylococcus aureus*	AMP, DA, ENR
387	*Staphylococcus aureus*	AMP, DA, TE, TOB, CN
T15	*Staphylococcus auricularis*	AMP
T32	*Staphylococcus capitis*	AMP
530 A	*Staphylococcus capitis*	AMP
T11	*Staphylococcus chromogenes*	DO, TOB, AK, RD
T3	*Staphylococcus epidermidis*	AMP, AMC, TOB
T22	*Staphylococcus epidermidis*	AMP, TOB, CN, SXT
T26	*Staphylococcus epidermidis*	AMP, DO, TE, TOB
T28	*Staphylococcus hominis*	AMP, DO, TE
T53	*Staphylococcus pseudointermedius*	DA
T33 P	*Staphylococcus lugdunensis*	DO, TOB, AK
T27	*Staphylococcus simulans*	AMP, DA, DO, TE, TOB, CN, AK, SXT, RD, ENR
T31 G	*Staphylococcus simulans*	AMP, DA, DO, TOB, CN, AK, SXT, RD, ENR
208 A	*Staphylococcus xylosus*	AMP, AMC, DA, DO, TE, TOB, CN, RD, ENR
234 2A	*Staphylococcus xylosus*	AMP, DO, TE, TOB, CN
T54	*Streptococcus constellatus*	TE

Legend. AK = amikacin; AMC = amoxycillin and clavulanic acid; AMP = ampicillin; DA = clindamycin; DO = doxycycline; ENR = enrofloxacin; CN = gentamicin; RD = rifampicin; SXT = trimethoprim–sulfamethoxazole; TE = tetracycline; and TOB = tobramycin.

Table S2. Antimicrobial resistance profiles for each examined Enterobacteriaceae isolate.

Identification number of the isolate	Bacterial species	Antimicrobial resistance profile
33 B	*Escherichia coli*	DA, RD
198	*Escherichia coli*	AMP, DA, DO, TE, CN, SXT, RD
502 B	*Escherichia coli*	DA, DO, TE, RD
856 A1	*Escherichia coli*	AMP, DA, SXT, RD
857 B1	*Escherichia coli*	AMP, DA, SXT, RD
858 A	*Escherichia coli*	AMP, DA, RD
220 B	*Klebsiella pneumoniae*	AMP, AMC, DA, DO, TE, RD
100	*Serratia marcescens*	AMP, AMC, DA, DO, TE, TOB, CN, SXT, RD, ENR

Legend. AK = amikacin; AMC = amoxycillin and clavulanic acid; AMP = ampicillin; DA = clindamycin; DO = doxycycline; ENR = enrofloxacin; CN = gentamicin; RD = rifampicin; SXT = trimethoprim–sulfamethoxazole; TE = tetracycline; and TOB = tobramycin.

Table S3. Antimicrobial resistance profiles for each examined *Pseudomonas aeruginosa* isolate.

Identification number of the isolate	Bacterial species	Antimicrobial resistance profile
178	*Pseudomonas aeruginosa*	AMP, AMC, DA, DO,TE, SXT, RD, ENR
348 B	*Pseudomonas aeruginosa*	AMP, AMC, DA, DO, TE, AK, SXT, RD
389	*Pseudomonas aeruginosa*	AMP, AMC, DA, DO, TE, SXT, RD, ENR
417	*Pseudomonas aeruginosa*	AMP, AMC, DA, DO, TE, SXT, RD, ENR
465	*Pseudomonas aeruginosa*	AMP, AMC, DA, DO, TE, CN, SXT, RD, ENR
502 A	*Pseudomonas aeruginosa*	AMP, AMC, DA, DO, TE, SXT, RD, ENR
535 A	*Pseudomonas aeruginosa*	AMP, AMC, DA, DO, TE, CN, AK, SXT, RD, ENR
768	*Pseudomonas aeruginosa*	AMP, AMC, DA, DO, TE, TOB, CN, AK, SXT, RD, ENR
822 A1	*Pseudomonas aeruginosa*	AMP, AMC, DA, DO, TE, TOB, SXT, RD, ENR
856 A2	*Pseudomonas aeruginosa*	AMP, AMC, DA, DO, TE, SXT, RD, ENR
857 B2	*Pseudomonas aeruginosa*	AMP, AMC, DA, DO, TE, SXT, RD, ENR
858 B	*Pseudomonas aeruginosa*	AMP, AMC, DA, DO, TE, SXT, RD
875 B1	*Pseudomonas aeruginosa*	AMP, AMC, DA, SXT, RD
876 A	*Pseudomonas aeruginosa*	AMP, AMC, DA, DO, TE, SXT, RD, ENR
876 B2	*Pseudomonas aeruginosa*	AMP, AMC, DA, DO, TE, TOB, AK, RD
1034	*Pseudomonas aeruginosa*	AMP, AMC, DA, DO, TE, CN, SXT, RD

Legend. AK = amikacin; AMC = amoxycillin and clavulanic acid; AMP = ampicillin; DA = clindamycin; DO = doxycycline; ENR = enrofloxacin; CN = gentamicin; RD = rifampicin; SXT = trimethoprim–sulfamethoxazole; TE = tetracycline; and TOB = tobramycin.
